# Neurotrophic Corneal Ulcer after Topical Tetracaine Abuse: Management Guidelines

**Published:** 2011-01-01

**Authors:** M R Sedaghat, S Sagheb Hosseinpoor, M Abrishami

**Affiliations:** 1 Department of Ophthalmology, Eye Research Center, School of Medicine, Mashhad University of Medical Sciences, Mashhad, Iran

**Keywords:** Neurotrophic Corneal Ulcer, Topical, Tetracaine, Abuse

**Fig. 1:**
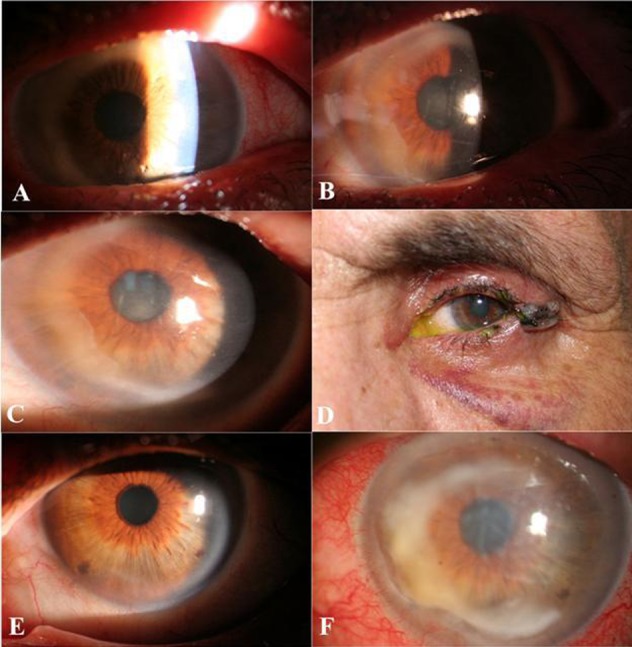
A) The first day: Ring infiltration, total epithelial defect, stromal edema, descemets folds of the cornea. B) 1 week after medication C) 3 weeks after medication: Decrease epithelial defect size but not healed totally. D) Tarsorrhaphy for epithelial healing is done. No epithelial defect and just small faint infiltration. E) After opening of the tarsorrhaphy: Clear cornea without any epithelial defect or infiltration. F) 2 years later: Ring infiltration, total epithelial defect, stromal edema, and descemets folds.

Dear Editor,

Indeed, topical ocular anesthesia has been part of modern ophthalmology for more than a century. All forms of anesthetics can be toxic, particularly when abused.[1] In Iran, the most common topical anesthetic is tetracaine. Most of the patients abuse it after UV keratitis or foreign body removal. The ocular surface and cornea are the most common sites of such toxicity. Systemic side effects are rare.[1][2][3] Unfortunately, it is growing as a health problem nowadays. Primary prevention is recommended by education of the workers to avoid abusing it. Here we report a one-eyed, 48-year-old man referred to us with an eye disorder from a few days ago following corneal foreign body removal. Corneal epithelial defect, ring infiltration, stromal edema, and descemets folds were observed at slit lamp examination (Figure 1A). In the history, he mentioned topical tetracaine use. He was asked to discontinue taking it. Ocular surface culture was performed but the result was negative. Topical autologus serum and oral steroid were administered. After one week, the size of epithelial defect was decreased (Figure 1B) at the third week, there was no significant change in epithelial defect size (Figure 1C). Lateral tarsorrhaphy was carried out (Figure 1D). One week later, the corneal epithelial defect was totally healed. After 2 months, the tarsorrhaphy was opened and the patient had clear cornea with no epithelial defect or infiltration (Figure 1E). Two years later, the patient returned with the same primary clinical feature (Figure 1F).

Topical anesthetics are over-the-counter drugs in many countries like Iran making it available for people, and increases the risk of its abuse. Symptoms of such patients included red eye, photophobia, decreased vision, ocular pain and tearing. Signs at the slit lamp exam are persistent corneal epithelial defect or neurotrophic ulcer, deep ring-shaped stromal infiltrate, folds in Descemet's membrane, anterior segment inflammation, ulceration and even perforation, dense cataract, corneal or scleral melting, secondary glaucoma and iritis.[2][3][4][5][6][8][9]

We have to take a careful history from the patient. In some cases it is also required to show the patients the anesthetic drugs and ask them about their consumption.[4][5][6][7] The drop was taken from the patient and was educated and warned about the hazards of its consumption. Next, the smear and the culture were provided to rule out the infectious etiology. In some cases, antibiotics are needed if an infectious cause is present.[2][3][4][5][6] Therefore we initiated a preservative free artificial tear therapy with lubricants. Adding topical autologous serum drop in the appropriate cases shows an interesting result in improving neurotrophic ulcers, like this one. In some cases, topical steroids, eye pressure patch, or soft contact lenses could also be beneficial.[2][5][6] Neurotrophic ulcer was shown to be caused due to a decrease in corneal sensitivity by an anesthetic abuse.[4][10] So we considered managements regarding neurotrophic ulcer treatment. We used oral corticosteroids after ruling out the infectious cause. When the response was insufficient, lateral tarsorrhaphy was performed as the next step. It has an effective role in improvement of persistent epithelial defects and prevention of disastrous complications like corneal perforation. In severe forms, penetrating keratoplasty may be needed [4][8] but fortunately, not in our patient. Psychiatric consultation and therapy are imperative in the management of some patients.[3] Functional and anatomic results after the appropriate treatment is not favorable in the majority of the cases, even leading to blindness3,8 But timely medication and interventions can survive the patient’s eye.

It is finally concluded that tetracaine abuse may cause neurotrophic ulcer and that the ulcer may be relapsed due to a repeat in the drug abuse. Use of oral corticosteroids and topical autologouse serums may play an important role in the treatment of such ulcers, but we cannot forget psychiatric consultation especially in prophylaxis of recurrence.
